# Prescription Pattern of Chinese Herbal Products for Diabetes Mellitus in Taiwan: A Population-Based Study

**DOI:** 10.1155/2013/201329

**Published:** 2013-06-17

**Authors:** Chung-Yu Huang, Yueh-Ting Tsai, Jung-Nien Lai, Feng-Lin Hsu

**Affiliations:** ^1^Institute of Traditional Medicine, School of Medicine, National Yang-Ming University, No. 155, Section 2, Linong Road, Taipei 112, Taiwan; ^2^Department of Traditional Medicine, Wan Fang Hospital, Taipei Medical University, Taipei 11696, Taiwan; ^3^Department of Chinese Medicine, Taipei City Hospital, Yangming Branch, Taipei 111, Taiwan; ^4^Graduate Institute of Pharmacognosy, College of Pharmacy, Taipei Medical University, No. 250, Wuxing Street, Taipei 11031, Taiwan

## Abstract

*Background.* Traditional Chinese medicine (TCM), when given as a therapy for symptom relief, has gained widespread popularity among diabetic patients. The aim of this study is to analyze the utilization of TCM among type 2 diabetic patients in Taiwan. *Methods.* The use of TCM for type 2 diabetic patients were evaluated using a randomly sampled cohort of 1,000,000 beneficiaries recruited from the National Health Insurance Research Database. *Results.* Overall, 77.9% (*n* = 31,289) of type 2 diabetic patients utilized TCM and 13.9% (*n* = 4,351) of them used TCM for the treatment of type 2 diabetes. Among the top ten most frequently prescribed herbal formulae, four remedies, *Zhi-Bo-Di-Huang-Wan, Qi-Ju-Di-Huang-Wan, Ji-Sheng-Shen-Qi-Wan* and *Ba-Wei-Di-Huang-Wan* are derivative formulae of *Liu-Wei-Di-Huang-Wan.* In other words, *Liu-Wei-Di-Huang-Wan* and its derivatives were found to be the most common herbal formulae prescribed by TCM doctors for the treatment of diabetes in Taiwan. *Conclusion.* Although some evidence does support the use TCM to treat diabetes, the results from the current study may have been confounded by placebo effect, which emphasize the need for well conducted, double-blind, randomized, placebo-controlled studies in order to further evaluate the efficacy of *Liu-Wei-Di-Huang-Wan* on patients with type 2 diabetes.

## 1. Introduction

Type 2 diabetes is becoming a pandemic disorder, and the related alarming increase in the prevalence of both microvascular and macrovascular disease has raised significant concerns [1–7]. Importantly, clinically significant morbidity is present at diagnosis, but the development of diabetes-related microvascular and macrovascular diseases may occur much earlier and well before diagnosis. Due to a lack of appropriate care for preclinical diabetes, diabetes expenditures have grown dramatically annually due to increased medical care required by patients with diabetes-related complications. Even worse, although many drugs improve glycemic control, they do not necessarily provide real-world benefits. Previous reports have indicated that combination therapy with metformin and glyburide and the use of thiazolidinediones increase the risk of a composite end point involving cardiovascular events and mortality [8, 9]. In addition, some diabetes medication unfortunately results in a number of common side effects such as nausea or upset stomach; these unwanted conditions drive patients to seek alternative advice [10]. Therefore, despite recent advances in intensive glycemic control, diabetes mellitus continues to be an important public health concern because it causes substantial morbidity and mortality as well as long-term complications [11, 12]. Not surprisingly, alternative therapies have become increasingly popular and are quickly approaching conventional therapy in their frequency of use as a treatment for diabetes and/or diabetes-related complications [13–15].

Previous studies of traditional Chinese medicines have found that *tianhuafen *(Radix Trichosanthis) [16]*, gegen *(Radix Puerariae) [17], *maimendong *(Radix Ophiopogonis) [18], *rougui *(Cortex Cinnamomi) [19, 20], *huangbai *(Cortex Phellodendri) [21] and *aconitum *(Aconitum carmichaeli) [22] may have antidiabetic activity, and *dihuang *(Radix Rehmanniae) [23]*, shanzhuyu *(Fructus Corni) [24], *renshen *(Radix Ginseng) [25], *gancao *(Radix Glycyrrhizae Praeparata) [26], and *bitter orange *(Aurantii Fructus) [27] have been suggested to increase insulin secretion. Unfortunately, evidence obtained in human studies is limited regarding patterns of use of classical traditional Chinese medicine (TCM) in relation to type 2 diabetes, which seem to be an area in which complementary and alternative medicines have recently grown in popularity. Furthermore, TCMs now seems to be marketed without established efficacy or safety in many Western countries [28, 29]. Owing to the previously and to a lack of knowledge about the prescription profile of TCMs, researchers and conventional doctors have found it difficult to explore the potential mechanisms of TCM therapies targeting type 2 diabetes. Furthermore, it also has proved difficult to assess the cost effectiveness of using TCM therapy and to observe the interaction between Chinese herbs and conventional diabetes drugs.

TCM, which includes acupuncture, traumatology manipulative therapies, and Chinese herbal products, has been an important part of health care in Taiwan for hundreds of years and is fully reimbursed under the current National Health Insurance (NHI) system. Previous studies using the NHI research database have reported that female [30] and individuals aged in their 30s [31] are more likely to use TCM among the general population in Taiwan. Chinese herbal remedies are the most common TCM modality used by this population, followed by acupuncture and traumatology manipulative therapies. The unique approach to TCM diagnosis involves gathering clinical symptoms and signs, and then a treatment principle is put forward in accordance with the aforementioned diagnostic process. In this situation, researchers in Taiwan have found that symptoms, signs, and ill-defined conditions are one of the most common reasons for TCM visits across various different patient populations [32–34]. Accordingly, the claims that database, part of the Taiwan National Health Insurance Research Database, provides are a platform for understanding the utilization of TCM therapies by licensed TCM doctors [30, 31, 35]. The aim of our study is to analyze a random sample from this comprehensive database and to determine the TCM utilization patterns of newly diagnosed type 2 diabetes patients in Taiwan. The results of this study should provide valuable information that will enable physicians to respond to patient use of TCM in an informed way, which will in turn strengthen further the patient-physician relationship when treating diabetes and diabetes-related complications. 

## 2. Materials and Methods

### 2.1. Data Resources

This study was designed as a population-based study analyzing a sample of one million subjects selected at random from the 22 million beneficiaries of the National Health Insurance scheme of Taiwan and aimed to determine the prevalence of using prescribed Chinese herbal prescriptions (CHP) among diabetes patients between January 1, 1998 and December 31, 2008. All data were obtained from the National Health Insurance Research Database (NHIRD), which includes all the reimbursement data of the NHI with the identification numbers of all individuals being encrypted and transformed; this database is maintained by the National Health Research Institutes of Taiwan [36]. The NHIRD database contains patient's gender and date of birth, all records of clinical visits and hospitalization, prescribed drugs and dosages, including CHP, and three major diagnoses coded in the *International Classification of Diseases, Ninth Revision,*  and  *Clinical Modification *(ICD-9-CM) formats [37]. 

### 2.2. Study Subjects

**Figure 1:**
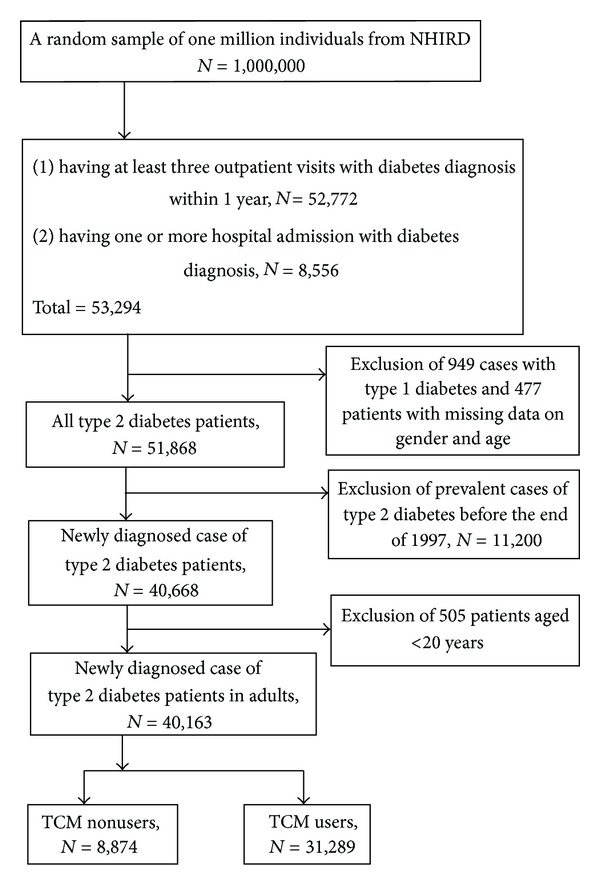
Flow recruitment chart of subjects from the one million random samples obtained from the National Health Insurance Research Database (NHIRD), 1997 to 2008, in Taiwan.

The selection of study subjects from the random sample of one million individuals was performed as follows (Figure 1). First, we included all patients that (1) had at least three outpatient visits with a diabetes diagnosis within 1 year (*n* = 52, 772) or (2) having one or more hospital admission with diabetes diagnosis (*n* = 8, 556) [38]. A total of 53,294 subjects were obtained. Second, we excluded all patients with type 1 diabetes (*n* = 949) or with missing information on gender and age (*n* = 477). Third, cases of diabetes (*n* = 11,200) that had been diagnosed before the end of 1997 were also excluded to ensure that all the subjects included were newly diagnosed with type 2 diabetes in the time period 1998–2008. Fourth, subjects under 20 years of age (*n* = 505) were also excluded to limit the study sample to adults. Finally, 40,163 study subjects were included in the study cohort. 

### 2.3. Study Variables

To determine the key independent variables for utilization of TCM among diabetes patients, we selected a series of demographic factors based on previous studies [3, 38–41]. The subjects were categorized into four groups according to age: 20–39, 40–59, 60–79, and ≥80 years. Taiwan was divided into seven geographic regions: Taipei city, Kaohsiung city, Northern region, Central region, Southern region, Eastern region and Outlying islands. We split the monthly wage of individuals into four levels: new Taiwan dollars (NT$) 0, 1–19,999, 20,000–39,999, and ≥40,000. We also searched the NHIRD database for clinical complications and treatment records related to diabetes as independent variables. The complications associated with diabetes included nephropathy (ICD-9 code, 581.81, 583.81, 585.1–585.9), retinopathy (362.01–362.07, 365.44, 366.41, 369.00–369.9), neuropathy (353.5, 536.3, 354.0–355.9, 713.5, 337.1, 357.2), peripheral circulatory disorders (250.70), and other specified manifestations (250.80) [42]. The reimbursement database contains all details related to the prescription of conventional medicines for treating diabetes mellitus. Then, for the final analysis, we categorized the types of preparations into the following categories: sulfonylureas, biguanides, *α*-glucosidase, meglitinide, thiazolidinediones, guar gum, and insulin. 

### 2.4. Statistical Analysis

Data analysis consisted of descriptive statistics, including the prescription rates of TCM users stratified by patient's demographic characteristics, indications for the prescription of TCM, and the most frequently prescribed herbal formulae used when treating diabetes. Primary indications were classified according to their ICD-9 code. The diagnoses were coded according to the ICD-9 and grouped into a series of distinct broad disease categories. The potential effects of Chinese herbs contained in the ten most commonly prescribed CHPs were grouped according to previous *in vivo *and *in vitro *studies and are summarized in Table 4 [16–27]. Multiple logistic regression was conducted to evaluate the factors that correlated with TCM use. A significance level of *α* = 0.05 was selected. The statistical software package SAS 9.13 was used for data management and analysis.

## 3. Results

The database of outpatient claims contained information on 40,163 patients with type 2 diabetes from 1998 to 2008. Among them, 31,289 (77.9%) patients used TCM outpatient services at least once. Most TCM users (91.2%) also received diabetes treatment. Among all TCM users, 13.9% (*n* = 4, 351) used TCM for the treatment of type 2 diabetes. Details of the demographic distribution of TCM users and nonusers are presented in Table 1. The mean age of TCM nonusers was slightly higher than that of TCM users. There were more TCM users than TCM nonusers with an income level of NT$ 20,000–39,999 or residing in Central Taiwan.

The adjusted odds ratios (aORs) and 95% confidence intervals (95% CIs) obtained by multiple logistic regression are also presented in Table 1. Compared with the age group 40–59 years (aOR = 1.00), those aged 20–39 years were more likely to be TCM users. There was also a significant difference between TCM users and nonusers with there being more of the former in the income group of NT$20,000–39,999. After adjusting for other factors, patients with more type 2 diabetes chronic complications (one complication: OR = 1.10, 95 %CI: 1.04–1.16; two complications: OR = 1.28, 95% CI: 1.19–1.38; more than three complications: OR = 1.25, 95% CI: 1.13–1.38) were more likely to seek TCM treatment than those with no chronic complication (aOR = 1.00). There was no significant difference in the diabetes treatment modalities received (monotherapy (aOR = 1.00) or combination therapy) between TCM users and TCM nonusers, except among those who took more than five types of antidiabetic drugs (OR = 1.13, 95% CI: 1.01–1.26) for the control of blood sugar or HbA1c.

Among the diabetes patients visiting TCM doctors, 3,627,622 (92.6%) visits involved the prescription of TCM, while the rest were prescribed acupuncture and traumatology manipulative therapies. Analysis of the major disease categories for all TCM visits made by 31,289 TCM users are summarized in Table 2. The findings show that “symptoms, signs, and ill-defined conditions” were the most common reason for using Chinese herbal prescriptions (CHP) (16.8%, *n* = 608,535), followed by “endocrine, nutritional and metabolic diseases, and immunity disorders” (12.1%, *n* = 439,612), and “diseases of digestive system” (11.5%, *n* = 417,611). Details of the most frequently prescribed CHP for treating type 2 diabetes by TCM doctors are provided in Table 3. *Liu-Wei-Di-Huang-Wan* (Rehmannia six pill) was the most frequently prescribed CHP, followed by *Bai-Hu-Jia-Ren-Shen-Tang *(white tiger plus ginseng combination),* Zhi-Bo-Di-Huang-Wan* (zhibai Rehmannia six pill),* Qi-Ju-Di-Huang-Wan *(chichu Rehmannia pill),* Yu-Quan-Wan* (jade spring pill),* Ji-Sheng-Shen-Qi-Wan *(economic health shenqi pill),* Xue-Fu-Zhu-Yu-Tang *(persica and achyranthes combination),* Ba-Wei-Di-Huang-Wan *(eight-flavour Rehmannia pill),* Bai-Hu-Tang* (white tiger combination), and *Gan-Lu-Yin *(sweet combination drink). Among the top ten most frequently prescribed herbal formulae, *Zhi-Bo-Di-Huang-Wan*,* Qi-Ju-Di-Huang-Wan*,* Ji-Sheng-Shen-Qi-Wan*, and* Ba-Wei-Di-Huang-Wan *are four derivative formulae of *Liu-Wei-Di-Huang-Wan*, which all contain Rhizoma Rehmanniae Preparata, Fructus Corni, Rhizoma Dioscoreae, Rhizoma Alismatis, Cortex Moutan Radicis, and Poria. In other words,* Liu-Wei-Di-Huang-Wan *and its various derivatives are the most common herbal formulae prescribed by TCM doctors for the treatment of diabetes in Taiwan. The ten most frequently prescribed CHPs include Chinese herbs that have been historically used to lower serum glucose. The potential effects of these Chinese herbs when used to treat type 2 diabetes are summarized in Table 4 and include increasing insulin secretion, enhancing glucose uptake by adipose and muscle tissues, inhibiting glucose absorption from intestine, inhibiting glucose production from hepatocytes, and decreasing insulin resistance or enhancing insulin sensitivity.

## 4. Discussion

The prevalence of type 2 diabetes in Taiwan over the 11 years in the study was 4.0%, which is in line with the estimates given by previous surveys [1, 43]. Worthy of note, the utilization of TCM among adults with type 2 diabetes in Taiwan during the study period was 77.9%, which appears to be high compared with previous findings [14, 15]. TCM is a unique traditional therapy approach for various ailments that has been used in Taiwan for over hundreds of years, and this long period of use may contribute significantly to the high prevalence of TCM usage among type 2 diabetic subjects. In addition, it should be noted that TCM treatment is covered by the NHI system. Therefore, unsurprisingly, the prevalence of CHP for treating type 2 diabetes among adults is comparatively higher in Taiwan than in other countries [15, 44]. The present study includes all patients who were newly diagnosed with type 2 diabetes by qualified conventional doctors between 1998 and 2008 from a random sample of one million subjects among the insured general population; importantly the rate of insured individuals has been consistently above 96% since 1997, and therefore we can rule out the possibility of selection bias.

The present results show that, although 91% of type 2 diabetic patients in Taiwan have received antidiabetic treatment, over half of them still have suffered from one or more diabetes complications during the 11-year follow-up. Nephropathy and neuropathy were the two most common diabetes complications. One possibility is that type 2 diabetes has a long asymptomatic preclinical phase that is likely to go undetected [45–49], and the injurious effects of asymptomatic hyperglycemia, therefore, have resulted in a high incidence of microvascular and macrovascular complications [4, 42]. The present study found that patients with type 2 diabetes who developed more than one site of involvement re-diabetes complications were more likely to seek advice from a TCM doctor. However, regardless of their experience in receiving more than one type of antidiabetic drug with the aim of improving their poor control of serum blood sugar, the choice of any of the major medical options available to patients with type 2 diabetes was not associated with the use of TCM. Hence, we suggest that when TCM is used to treat type 2 diabetes in Taiwan, this is generally an adjunct to diabetes treatment rather than a replacement for it. 

The present findings show that, among diabetes patients, females and those aged 20–39 years were more likely to be TCM users than males and other age groups as shown in Table 1. As shown in Table 2, “symptoms, signs, and ill-defined conditions” were the most common reasons for using CHP (16.8%, *n* = 608,535), followed by “endocrine, nutritional and metabolic diseases, and immunity disorders” (12.1%, *n* = 439,612) and “diseases of digestive system” (11.5%, *n* = 417, 611). Further analysis found that TCM doctors tended to use Chinese herbal remedies targeting diabetes as well as gastrointestinal disorders that might be the uncomfortable side effects of diabetes drugs. Although previous studies have demonstrated that acupuncture might be related to an alternative therapy for treating hyperglycemia and diabetes complications, the present study indicated that acupuncture in Taiwan is used by this study population mainly for diseases of the musculoskeletal system and connective tissue.

*Liu-Wei-Di-Huang-Wan *was the most frequently prescribed formula for treating type 2 diabetes in Taiwan during the study period, as shown in Table 3. *Liu-Wei-Di-Huang-Wan* is among the most highly regarded ancient Chinese herbal formulae and was first documented in the classical Chinese text *Xiao Er Yao Zheng Zhi Jue *(Key to Therapeutics of Children's Diseases) circa 1119 A.D. In the classical literature, *Liu-Wei-Di-Huang-Wan* is said to nourish yin and to invigorate the kidney, which might indicate it as a potentially efficacious therapy for reducing hyperglycemia and relieving neuropathic and nephropathic complications in diabetes mellitus [50–53]. Among the top ten most frequently prescribed formulae for treating type 2 diabetes, *Zhi-Bo-Di-Huang-Wan*, *Qi-Ju-Di-Huang-Wan*,* Ji-Sheng-Shen-Qi-Wan*, and *Ba-Wei-Di-Huang-Wan*, which are all derivatives of *Liu-Wei-Di-Huang-Wan*, are prescribed to alleviate various common symptoms of type 2 diabetes, namely, unusual thirst, blurred vision, frequent urination, and cold feeling in the limbs, respectively. Other frequently prescribed formulae are associated with severe dysphoric thirst and lassitude (*Bai-Hu-Jia-Ren-Shen-Tang *or white tiger plus ginseng decoction, *Bai-Hu-Tang *or white tiger decoction,* Yu-Quan-Wan *or jade spring combination, and *Gan-Lu-Yin* or sweet dew decoction or sweet combination drink) and with peripheral neuropathy due to blood stasis (*Xue-Fu-Zhu-Yu-Tang *or Persica and Carthamus Combination). Although, previous *in vitro* studies have found that some Chinese herbs are able to decrease serum levels of glucose, glycosylated proteins, and hemoglobin A1C, possibly by blocking intestinal absorption and/or inhibiting hepatic glucose-6-phosphatase [50, 54], there have not yet been any clinical trials that have demonstrated the efficacy and safety of *Liu-Wei-Di-Huang-Wan *and its derivatives when treating diabetes type 2. In general, TCM doctors treated diabetes patients' complaints according to the syndrome differentiation theory rather than by making a specific diagnosis; this is based on holistic consideration of diabetes patients who are suffering from symptoms and complications at various sites. In this context and in line with previous results [51], the present study found that TCM doctors in Taiwan prescribed herbal therapies mainly to optimize the body's ability to function normally, rather than as a cure for diabetes. Moreover, despite inadequate data on the clinical safety and efficacy of CHP when treating diabetic patients, a large number of patients use them. Thus, based on the present trend in TCM utilization, herbal remedies for treating diabetes and/or diabetic complications will continue to be used. Although we respect the patients' choice of medical care, we recommend that TCM practitioners and physicians should carefully monitor patient blood glucose levels and the potential side effects of CHP when they are being used alongside or in lieu of diabetes drugs. Further studies are warranted to assess the formulae generally used by TCM doctors in this study in order to determine whether they are really useful as add-on treatments for patients receiving antidiabetic treatment.

The present study has three limitations. First, this study did not include Chinese herbal remedies or decoctions that were purchased directly from TCM herbal pharmacies, nor did we include health foods containing herbs. Thus, the frequency of CHP utilization might have been underestimated. However, because the NHI system covers TCM prescriptions, which generally cost less than the herbs sold in Taiwan's markets, the likelihood that subjects purchased a lot of other herbs outside the NHI database is not high. Second, we are unable to draw any conclusion about the relationship of blood glucose and haemoglobin A1C levels with respect to TCM utilization owing to the lack of actual clinical data. Third, this was a retrospective study and thus does not include a randomized placebo group. Thus, great caution is necessary when interpreting the results of the most commonly prescribed Chinese formulae obtained in the present study due to the possibility of a placebo effect.

## 5. Conclusions

Our results suggest that, based on the coexistence of both conventional and traditional Chinese medical treatments, of most the diabetes patients consume herbal therapies with the intention of relieving their diabetes-related symptoms, rather than because they have rejected standard diabetes treatments. *Liu-Wei-Di-Huang-Wan *and its derivatives are the most frequently prescribed formulae by TCM doctors in Taiwan for diabetes patients. Having recognized the use of TCM, exploring any potential interactions and adverse effects, and integrating both technologies into a holistic treatment system may be beneficial to the overall health, presence of comorbidities, and quality of life, of patients with type 2 diabetes. It is worth noting that although some evidence does support the use of TCM to treat diabetes, the results from the current study may have been confounded by the placebo effect. This emphasizes the need for well-conducted, double-blind, randomized, placebo-control studies to further evaluate efficacy when *Liu-Wei-Di-Huang-Wan *is given to patients with type 2 diabetes.

## Figures and Tables

**Table 1:** Demographic characteristics and results of multiple logistic regressions showing the adjusted odds ratio (aOR) and 95% CI (confidence interval) for diabetes from 1998 to 2008 in Taiwan.

Characteristic	All patients	TCM^a^ nonusers (%)	TCM users (%)	aOR^b^ (95% CI^c^)
Number of cases	40,163	8,874	31,289	
Gender				
Male	20,971 (52.2)	5,718 (64.4)	15,253 (48.7)	1.00
Female	19,192 (47.8)	3,156 (35.6)	16,036 (51.3)	1.98 (1.88–2.08)
Age at diagnosis (years)				
Mean ± SD	56.7 ± 12.4	58.3 ± 12.9	56.3 ± 12.2	
20~39	3,175 (7.9)	609 (6.9)	2,566 (8.2)	1.11 (1.01–1.22)
40~59	20,523 (51.1)	4,184 (47.1)	16,339 (52.2)	1.00
60~79	15,244 (38.0)	3,653 (41.2)	11,591 (37.0)	0.79 (0.75–0.83)
≥80	1,221 (3.0)	428 (4.8)	793 (2.5)	0.46 (0.41–0.52)
Insured salaries (NT$^d^/month)				
0	9,981 (24.9)	2,207 (24.9)	7,774 (24.9)	1.00
1–19,999	21,256 (52.9)	4,801 (54.1)	16,455 (52.6)	1.01 (0.95–1.08)
20,000–39,999	5,414 (13.5)	982 (11.1)	4,432 (14.2)	1.33 (1.21–1.46)
≥40,000	3,512 (8.7)	884 (9.9)	2,628 (8.4)	1.02 (0.92–1.12)
Insured region				
Taipei city	6,975 (17.4)	1,746 (19.7)	5,229 (16.7)	1.00
Kaohsiung city	2,802 (7.0)	606 (6.8)	2,196 (7.0)	1.21 (1.09–1.35)
Northern Taiwan	11,316 (28.2)	2,652 (29.9)	8,664 (27.7)	1.10 (1.02–1.18)
Central Taiwan	7,172 (17.8)	1,070 (12.1)	6,102 (19.5)	1.92 (1.76–2.09)
Southern Taiwan	10,447 (26.0)	2,436 (27.4)	8,011 (25.6)	1.12 (1.04–1.20)
Eastern Taiwan	1,130 (2.8)	288 (3.3)	842 (2.7)	0.97 (0.83–1.12)
Outlying islands	319 (0.8)	75 (0.8)	244 (0.8)	1.11 (0.85–1.45)
Number of diabetic complications				
0	17,913 (44.6)	4,213 (47.5)	13,700 (43.8)	1.00
1	12,833 (32.0)	2,823 (31.8)	10,010 (32.0)	1.10 (1.04–1.16)
Nephropathy	2,990 (7.4)	724 (8.1)	2,266 (7.2)	
Retinopathy	2,764 (6.9)	674 (7.6)	2,090 (6.7)	
Neuropathy	5,339 (13.3)	993 (11.2)	4,346 (13.9)	
Peripheral circulatory disorders (PCD)	844 (2.1)	213 (2.4)	631 (2.0)	
Other specified manifestations	896 (2.2)	219 (2.5)	677 (2.2)	
2	6,296 (15.7)	1,226 (13.8)	5,070 (16.2)	1.28 (1.19–1.38)
Nephropathy + retinopathy	1,039 (2.6)	241 (2.7)	798 (2.5)	
Nephropathy + neuropathy	1,588 (4.0)	300 (3.4)	1,288 (4.1)	
Nephropathy + PCD	256 (0.6)	64 (0.7)	192 (0.6)	
Nephropathy + others	273 (0.7)	61 (0.7)	212 (0.7)	
Retinopathy + neuropathy	1,579 (3.9)	250 (2.8)	1,329 (4.3)	
Retinopathy + PCD	234 (0.6)	45 (0.5)	189 (0.6)	
Retinopathy + others	206 (0.5)	46 (0.5)	160 (0.5)	
Neuropathy + PCD	582 (1.4)	107 (1.2)	475 (1.5)	
Neuropathy l + others	429 (1.1)	81 (0.9)	348 (1.1)	
PCD + others	110 (0.3)	31 (0.4)	79 (0.3)	
≥3	3,121 (7.7)	612 (6.9)	2,509 (8.0)	1.25 (1.13–1.38)
Number of medical treatments for diabetes				
None	3,370 (8.4)	627 (7.1)	2,743 (8.8)	
Monotherapy^e^	6,048 (15.1)	1,443 (16.3)	4,605 (14.7)	1.00
Two-drug combination^f^	13,609 (33.9)	3,127 (35.2)	10,482 (33.5)	1.01 (0.94–1.09)
Three-drug combination	8,675 (21.6)	1,937 (21.8)	6,738 (21.5)	1.00 (0.93–1.09)
Four-drug combination	5,026 (12.5)	1,093 (12.3)	3,933 (12.6)	1.01 (0.92–1.10)
Over five-drug combination	3,435 (8.5)	647 (7.3)	2,788 (8.9)	1.13 (1.01–1.26)

^a^TCM refers to traditional Chinese medicine; ^b^OR refer to odds ratio; ^c^CI refers to confidence interval; ^d^NT$ refers to new Taiwan dollars, of which US$ 1 = NT$30 approximately.

^e^Monotherapy is the use of a single antidiabetic drug (sulfonylureas, biguanides, *α*-glucosidase, meglitinide, thiazolidinediones, guar gum, or insulin).

^f^combination therapy is the use of more than one antidiabetic drug.

**Table 2:** Frequency distribution of traditional Chinese medicine (TCM) visits by major disease categories (according to 9th ICD codes) among diabetes patients from 1998 to 2008 in Taiwan.

Major disease category	ICD-9-CM codes	No. of visits (no. of patients)		
		Chinese herbal remedies	Acupuncture or manipulative therapies	Total of TCM
Infectious and parasitic diseases	001–139	21,083 (761)	69 (13)	21,152 (772)
Neoplasms	140–239	33,132 (366)	613 (20)	33,745 (381)
Endocrine, nutritional and metabolic diseases, and immunity disorders	240–279	439,612 (5,565)	1,995 (127)	441,607 (5,620)
Diabetes	250	377,621 (4,328)	1,505 (65)	379,126 (4,350)
Others		61,991 (1,703)	490 (62)	62,481 (1,742)
Mental disorders	290–319	24,834 (858)	536 (28)	25,370 (876)
Diseases of nervous system and sense organs	320–389	104,033 (3,962)	4,593 (802)	108,626 (4,513)
Diseases of circulatory system	390–459	180,821 (3,647)	5,857 (444)	186,678 (3,892)
Diseases of respiratory system	460–519	377,262 (10,505)	1,423 (126)	378,685 (10,537)
Diseases of digestive system	520–579	417,611 (9,387)	1,332 (129)	418,943 (9,432)
Diseases of genitourinary system	580–629	171,262 (4,145)	1,294 (63)	172,556 (4,170)
Diseases of skin and subcutaneous tissue	680–709	61,387 (2,784)	280 (41)	61,667 (2,810)
Diseases of musculoskeletal system and connective tissue	710–739	318,920 (8,808)	82,936 (12,682)	401,856 (16,932)
Symptoms, signs, and ill-defined conditions	780–799	608,535 (14,216)	3,839 (458)	612,374 (14,345)
Injury and poisoning	800–999	17,994 (1,389)	90,542 (14,141)	108,536 (14,625)
Supplementary classification^+^	V01–V82,	115 (9)	0 (0)	115 (9)
	E800–E999	0 (0)	0 (0)	0 (0)
Others*		851,021 (15,034)	96,311 (11,164)	947,332 (18,954)

Total		3,627,622 (27,135)	291,620 (22,891)	3,919,242 (31,289)

*Others include ICD-9-CM codes 280–289, *630–677, 740–759, *760–779 and missing/error data; ^+^Supplementary classification of factors influencing health status and contact with health service, external causes of injury and poisoning.

**Table 3:** Ten most common herbal formulae prescribed by TCM doctors for the treatment of type 2 diabetes among 31,289 patients from 1998 to 2008 in Taiwan.

Herbal formulae	English name	Number of person-days *N* = 775,447 (%)	Average daily dose (g)	Average duration for prescription (days)
*Liu-Wei-Di-Huang-Wan *	Rehmannia six pill	62,249 (8.0)	8.0	47.9
*Bai-Hu-Jia-Ren-Shen-Tang *	White tiger plus ginseng combination	42,676 (5.5)	7.5	47.4
*Zhi-Bo-Di-Huang-Wan *	Zhibai Rehmannia six pill	37,918 (4.9)	5.7	45.4
*Qi-Ju-Di-Huang-Wan *	Chichu Rehmannia pill	37,796 (4.9)	5.7	64.4
*Yu-Quan-Wan *	Jade spring pill	35,878 (4.6)	5.6	54.8
*Ji-Sheng-Shen-Qi-Wan *	Economic health shenqi pill, life-saving renal Chi pill	27,347 (3.5)	7.1	43.6
*Xue-Fu-Zhu-Yu-Tang *	Persica and achyranthes combination	22,708 (2.9)	5.3	45.9
*Ba-Wei-Di-Huang-Wan *	Eight-flavour Rehmannia pill	19,247 (2.5)	9.1	41.5
*Bai-Hu-Tang *	White tiger combination	18,801 (2.4)	7.0	42.1
*Gan-Lu-Yin *	Sweet combination drink	18,502 (2.4)	5.1	38.1

**Table 4:** Potential effects of herbs present in the ten most common herbal formulae prescribed by TCM doctors for treating type 2 diabetes.

Herbal formulae	Number of herbs	Ingredient herbs
*Liu-Wei-Di-Huang-Wan *	6	Rhizoma Rehmanniae Praeparata^A,B,D,E^, Fructus Corni^A,D^, Rhizoma Dioscoreae^B,E^, Rhizoma Alismatis^B^, Cortex Moutan Radicis, Poria^B,E^.
*Bai-Hu-Jia-Ren-Shen-Tang *	5	Gypsum Fibrosum, Rhizoma Anemarrhenae^E^, Radix Glycyrrhizae Praeparata^A^, Semen Oryzae Sativae, Radix Ginseng^A,B,C,D^.
*Zhi-Bo-Di-Huang-Wan *	8	Rhizoma Anemarrhenae^E^, Cortex Phellodendri, Rhizoma Rehmanniae Praeparata^A,B,D,E^, Fructus Corni^A,D^, Rhizoma Dioscoreae^B,E^, Rhizoma Alismatis^B^, Cortex Moutan Radicis, Poria^B,E^.
*Qi-Ju-Di-Huang-Wan *	8	Flos Chrysanthemi, Fructus Lycii^B,E^, Rhizoma Rehmanniae Praeparata^A,B,D,E^, Fructus Corni^A,D^, Rhizoma Dioscoreae, Rhizoma Alismatis^B^, Cortex Moutan Radicis, Poria^B,E^.
*Yu-Quan-Wan *	9	Radix Trichosanthis, Radix Puerariae^A,B^, Radix Ophiopogonis^A,C,D^, Radix Ginseng, Poria^B,E^, Radix Astragali^B,E^, Radix Glycyrrhizae Praeparata, Fructus Mume, Radix Astragali Praeparata.
*Ji-Sheng-Shen-Qi-Wan *	10	Semen Plantaginis, Radix Achyranthis Bidentatae, Ramulus Cinnamomi, Radix Aconiti, Rhizoma Rehmanniae Praeparata, Fructus Corni, Rhizoma Dioscoreae^B,E^, Rhizoma Alismatis^B^, Cortex Moutan Radicis, Poria^B,E^.
*Xue-Fu-Zhu-Yu-Tang *	11	Chinese Angelia Root, Rhizoma Rehmanniae Praeparata, Peach Kernel, Safflower, Bitter Orange^A^, Red Peony Root, Bupleurum Root, Glycyrrhiza, Platycodon Root, Chuanxiong Rhizome, Cyathula Root.
*Ba-Wei-Di-Huang-Wan *	8	Ramulus Cinnamomi^E^, Radix Aconiti^B^, Rhizoma Rehmanniae Praeparata, Fructus Corni, Rhizoma Dioscoreae, Rhizoma Alismatis, Cortex Moutan Radicis, Poria^B,E^.
*Bai-Hu-Tang *	4	Gypsum Fibrosum, Rhizoma Anemarrhenae, Radix Glycyrrhizae Praeparata, Semen Oryzae Sativae.
*Gan-Lu-Yin *	10	Rhizoma Rehmanniae, Radix Ophiopogonis^A,C,D^, Radix Glycyrrhizae Praeparata, Herba Dendrobii, Radix Asparagi, Eriobotryae Folium, Bitter Orange^A^, Scutellariae radix, Wormwood Herb, Rhizoma Rehmanniae Praeparata.

^A^Increase in insulin secretion, ^B^enhancement of glucose uptake by adipose and muscle tissues, ^C^inhibition of glucose absorption by the intestine, ^D^inhibition of glucose production by hepatocytes, and ^E^decrease in insulin resistance or enhancement of insulin sensitivity.
